# Adolescent, parent, and provider attitudes toward a machine-learning based clinical decision support system for selecting treatment for youth depression

**DOI:** 10.21203/rs.3.rs-3374103/v1

**Published:** 2023-10-03

**Authors:** Meredith Gunlicks-Stoessel, Yangchenchen Liu, Catherine Parkhill, Nicole Morrell, Mimi Choy-Brown, Christopher Mehus, Joel Hetler, Gerald August

**Affiliations:** University of Minnesota

**Keywords:** clinical decision support systems, depression, adolescents, health care providers, attitudes

## Abstract

**Background:**

Machine-learning based clinical decision support systems (CDSSs) have been proposed as a means of advancing personalized treatment planning for disorders, such as depression, that have a multifaceted etiology, course, and symptom profile. However, machine-learning based models for treatment selection are rare in the field of psychiatry. They have also not yet been translated for use in clinical practice. Understanding key stakeholder attitudes toward machine learning-based CDSSs is critical for developing plans for their implementation that promote uptake by both providers and families.

**Methods:**

In Study 1, a machine-learning based Clinical Decision Support System for Youth Depression (CDSS-YD) was demonstrated to focus groups of adolescents with a diagnosis of depression (n = 9), parents (n = 11), and behavioral health providers (n = 8). Qualitative analysis was used to assess their attitudes towards the CDSS-YD. In Study 2, behavioral health providers were trained in the use of the CDSS-YD and they utilized the CDSS-YD in a clinical encounter with 6 adolescents and their parents as part of their treatment planning discussion. Following the appointment, providers, parents, and adolescents completed a survey about their attitudes regarding the use of the CDSS-YD.

**Results:**

All stakeholder groups viewed the CDSS-YD as an easy to understand and useful tool for making personalized treatment decisions, and families and providers were able to successfully use the CDSS-YD in clinical encounters. Parents and adolescents viewed their providers as having a critical role in the use the CDSS-YD, and this had implications for the perceived trustworthiness of the CDSS-YD. Providers reported that clinic productivity metrics would be the primary barrier to CDSS-YD implementation, with the creation of protected time for training, preparation, and use as a key facilitator.

**Conclusions:**

The CDSS-YD has the potential to be a widely accepted and useful tool for personalized treatment planning. Successful implementation will require addressing the system-level barrier of having sufficient time and energy to integrate it into practice.

## Introduction

Depression among adolescents is becoming an increasingly critical public health concern. An estimated 4.1 million adolescents in the United States had at least one major depressive episode in 2020 (1). This represents 17.0% of the population of 12–17 year-olds and is an increase from 11.3% in 2014 and 8.7% in 2005 (1, 2). The rise in prevalence is compounded by the fact that while treatment options exist, 30–50% of youth who receive an evidence-based treatment do not experience the intended reduction in symptoms (3, 4).

Mental health experts have proposed that because depression is a disorder that is characterized by a multifaceted etiology, course, and symptom profile, treating it effectively may require careful consideration of each patient’s unique characteristics to inform the treatment selection and matching process (5, 6). This has been the promise of the precision medicine movement which aims to identify key markers of treatment response that can inform personalized treatment planning. However, given the complexity of depression, individual markers likely only provide partial information regarding a patient’s expected treatment response. Developing more precise, and potentially more effective guidelines requires computational modeling techniques that can examine a large number of markers simultaneously as potential predictors of treatment outcome. Machine-learning methods, which can be used for predictive modeling from high dimensional, multi-modular data, are well suited for treatment outcome estimation (7, 8). These methods are beginning to be utilized to successfully construct high quality models for diagnosis, prognosis, and treatment assignment in mental health and other fields of medicine (9–12). However, these models are rare, and to our knowledge, have not yet been developed for treatment selection for youth with depression. They have also not yet been translated for use in clinical practice.

Our team applied machine learning methods to develop a treatment selection algorithm that provides a treatment recommendation for adolescents with depression based on data spanning a range of clinical and psychosocial domains from the Treatment for Adolescents with Depression Study (TADS) (4). The model identified subgroups of adolescents who respond differentially to cognitive behavioral therapy (CBT), medication, and their combination, with effect sizes in the large range (Authors, 2020). We have built the algorithm in a digital web-based platform to create a CDSS that can provide patient-specific treatment recommendations that are delivered at the point of care. The Clinical Decision Support System for Youth Depression (CDSS-YD) includes five components, all of which were built in the web-based system Research Electronic Data Capture (REDCap): (1) self-report questionnaires completed by the adolescent prior to the clinical appointment; (2) questionnaires completed by the parent prior to the appointment; (3) semi-structured interviews conducted by a mental health provider with the adolescent and the parent during the appointment; (4) an algorithm that computes the adolescents’ predicted depression outcome with CBT, fluoxetine, and combination treatment based on the completed measures; and (5) a treatment recommendation page that displays the treatment(s) recommended by the algorithm, as well as the scores on the measures completed by the adolescent, parent, and provider.

CDSSs have been used in other domains of medicine, but their use in psychiatric care is rare. Studies show that CDSSs improve physician performance and patient treatment outcomes (13). However, a recent meta-analysis of CDSS trials found that CDSS uptake by physicians was low (14). Provider attitudes towards CDSSs have been identified a key barrier to CDSS use. Identified attitudinal barriers include beliefs that the use of CDSSs may reduce their professional autonomy, provide unneeded guidance, interfere with the provider-patient therapeutic relationship, or be used against them in the case of medical or legal controversies (15, 16). This highlights the critical need to identify and address provider attitudes for CDSS uptake to be successful. Patient attitudes are also critical, as CDSS use is also predicted by availability and quality of the patient data needed to inform the CDSS (14). In the field of mental health, this data often comes from questionnaires that are completed by the patients themselves. Patient attitudes towards the completion of questionnaires are likely to impact whether and how they complete them. In addition, given that treatment planning should be a collaborative process in which the provider and patient work together to engage in shared decision making (17), patient attitudes towards the CDSS and its treatment recommendations would also be expected to impact CDSS use. Shared decision in youth mental health care also has the additional complexity of needing to incorporate the attitudes of both youth and their caregivers.

Understanding key stakeholder attitudes toward CDSSs, including potential barriers and facilitators to their use, is critical for developing CDSSs and plans for their implementation that promote uptake by both providers and families. CDSSs based on machine learning-derived algorithms have the potential to elicit some unique attitudes from providers and families alike, as the treatment recommendations are derived from a complex statistical model as opposed to a more comprehensible guideline, such as one based on depression severity. To our knowledge, there is currently no other research that has evaluated provider and patient attitudes towards a machine learning-based CDSS. The goal of the current study was to collect feedback from youth, parents, and providers on the CDSS-YD. In Study 1, we demonstrated the CDSS-YD to focus groups of adolescents, parents, and behavioral health providers and elicited their feedback on 1) the acceptability and appropriateness of the CDSS-YD, 2) determinants of CDSS-YD use, and 3) potential impact of the CDSS-YD on treatment processes. In Study 2, a small sample of adolescents with a diagnosis of a depressive disorder and their parents utilized the CDSS-YD with their behavioral health provider during a clinical encounter as part of a treatment planning discussion, and they shared their experience of using the CDSS-YD.

## Study 1

### Methods

#### Participants.

Adolescents and parents/caregivers were recruited from a clinical trial of treatments for depression in adolescents conducted by the principal investigator. Families were contacted if they had provided consent to be contacted about future research opportunities. Nine adolescents (age range = 13–16, mean age = 15.11, SD = 1.05) participated in the focus groups. Seven adolescents reported their sex assigned at birth as female and two reported their sex as male. Gender identity was as follows: four female, two male, one genderqueer/gender nonconforming/neither, and two other. Six of the adolescents identified as White (67%), four identified as Black/African American (44%), three identified as Asian (33%), and one preferred not to answer. Eleven caregivers participated in this study (nine biological mothers and two grandmothers with legal guardianship). The mean age of caregivers was 48.09 (SD=10.60), and their race was White (82%), Black/African American (9%), and Asian (9%).

Behavioral health providers who work with adolescent-age patients were recruited from two services within a non-profit, integrated health care system. Eight master’s level therapists (LMFT, MSW) participated in the study. Four therapists were co-located in primary care clinics to provide assessment and treatment referral for youth who were identified by a primary care provider as needing behavioral health care, and four therapists were outpatient therapy providers in the health care system’s counseling centers. All therapists identified sex assigned at birth and gender as female. All therapists identified as White and one identified as Hispanic/Latino.

#### Study Design.

A total of six focus groups were conducted: two with adolescents, two with parents, and two with providers. Semi-structured interview guides for each group were designed by the research team and guided by relevant constructs from the literature on CDSS implementation in other fields. The primary domains of interest were perspectives on 1) the acceptability and appropriateness of the CDSS-YD, 2) determinants of use of the CDSS-YD, and 3) potential impact on treatment processes.

The provider focus group protocol was assessed by the institution’s IRB to be Not Human Research. As a consequence, providers provided verbal consent prior to participation, but did not provide written documentation of consent. Adolescent and parent/caregiver participants provided signed informed consent and assent prior to completing research procedures.

Each focus group was facilitated by two members of the research team. The primary group leader introduced the CDSS-YD to participants, then asked their feedback on it, following the drafted interview guides. Stakeholder groups were asked about 1) the acceptability and appropriateness of the CDSS-YD (e.g. “To what extent do you understand the information provided in the CDSS-YD?”, “How useful is the CDSS-YD for making a treatment decision?”), 2) determinants of CDSS-YD use (e.g. “What would make this tool more usable/user-friendly in practice?”, “What would make you more likely to actually use this?”, “What do you think would help make sure teens/parents complete the questionnaires prior to the appointment?”), and 3) potential impact of the CDSS-YD on treatment processes (e.g. “How do you think you using the CDSS-YD would impact your trust in the treatment recommendation?”, “How do you think using a decision guide like this would impact how much you feel involved in the treatment planning process?”, “To what extent do you feel like using a decision guide like this would impact your relationship with your treatment provider?”). All focus-group interviews were conducted virtually over a web-based video platform. The focus groups were video-recorded, and the audio files were transcribed.

#### Analysis.

Data was coded using a thematic analysis approach (18). The initial codebook was generated from domains of interest and emergent codes from the transcripts. The final codebook was finalized through consensus between two coders. Next, the two coders returned to the data and independently coded the transcripts. Disagreements between coders were resolved through discussion and consensus.

### Results

#### Attitudes Toward the CDSS-YD.

Key themes, subthemes, and exemplar quotations are displayed in Table 1. Five key themes were found: (1) providers, parents, and adolescents viewed the CDSS-YD as helpful for treatment; (2) providers, parents, and adolescents differed in their views of the trustworthiness of the CDSS-YD, with adults having more trust in the data-driven approach of the CDSS-YD and adolescents having more trust in the provider’s expertise; (3) parents and adolescents saw their providers as having a critical role in the use the CDSS-YD; (4) providers, parents, and adolescents expressed a desire to understand how the questionnaire responses informed the CDSS-YD’s treatment recommendation; and (5) adolescents expressed discomfort with sharing their questionnaire results with the parents, and they expressed a desire for privacy when reviewing the CDSS-YD results with the provider.

#### Attitudes Regarding Barriers and Facilitators of CDSS-YD Implementation.

Providers reported that the primary barrier to CDSS-YD implementation was clinic productivity metrics (“Thaťs a big thing with us, the productivity. Everything’s numbers, numbers, numbers, numbers, numbers. And if you miss an hour, where are you going to make it up? And we canť make it up because we’re full. There’s no other place to put people.”). The pressure to maximize productivity had downstream perceived negative impacts on CDSS-YD implementation, as well. These included provider burnout (“There’s a lot of burnout right now.” “My co-workers would say that iťs too much stuff on top of what we’re already asked to do.”), and inadequate time (“Anything to make the process work better for our clients and work better for our families, I think we naturally are on board for those types of things, but iťs hard. When and where do we do this? When and where do we even have time to review some of the stuff before our sessions or after our sessions when there’s so much. So iťs kind of, how can we juggle? How can we juggle it.”). They also noted that lack of integration with the electronic medical record would be a barrier, due to the additional time that would be involved in using a separate system (“They wonť use it if iťs not in the medical record. If iťs not in front of them, they’re… I mean, we have providers that donť even check emails honestly… So if iťs not in front of them, iťs not going to do anything.”)

The pressure of productivity and its impact on time-limited clinical workflow also had a perceived negative impact on families’ completion of the questionnaires that are essential for CDSS-YD use (“I feel like half the time with the assessments that they’re usually not done before the visit. And then that takes up a chunk of our time in the visit, which is frustrating.”, “There’s a ton of things [that are part of the intake process]. So they’re exhausted, I’m exhausted, by the time we’ve gotten done. So now we’re adding that piece. It could be a lot.”).

Providers reported that the primary facilitator to using CDSS-YD would be protected time for training, preparation, and use (“I would think I would probably need like two documentation times that they’re not going to get filled. Thaťs going to be able to be devoted to what I need to do to learn this, implement it properly, be able to work with it. Kind of block times in our schedule that we’re guaranteed are not going to get a client put in there. We’re getting productivity for that hour.”). This protected time included having advanced notice that the clinic would be adopting a new clinical workflow (“Biggest thing for me would be ample time talking about it ahead of time. Because a lot of times our roles are, ‘by the way today we’re starting this.’ Maybe having a conversation in, like, consult group, what the benefit is of it versus it being this email that comes from nowhere and is pretty immediate.”).

Protected time was also viewed as a facilitator to families’ successful completion of the questionnaires needed for CDSS-YD use. For example, parents and adolescents reported that scheduling families to come to their appointment early to complete the questionnaires while waiting for their provider would be helpful (“I might say just like to actually do it at the appointment just because it’s easy to forget about stuff like that. So if there’s an actual scheduled time to get it done, I think that might be easier.” – adolescent). Taking the time to explain to families why it is important to complete the questionnaires was also viewed as critical (“Like if you’re making that appointment and they’re like, ‘okay, we have these questionnaires,’ and explaining why iťs super important to answer them and take the time to answer them rather than just, okay, ‘I’ll send you some stuff and fill it out.’” – parent).

An additional facilitator of CDSS-YD use that was identified by providers was positive messaging. They reported that hearing positive feedback from co-workers and families would increase their likelihood of wanting to use the CDSS-YD (“I would say hearing success stories of it being used and the benefit of it being used and actual scenarios that happen in the clinics. I think that is one of the best ways.”). They also reported that the use of clinic champions would be helpful (“It may be too, that if we struggle with getting enough people to buy in that the few of us that do it can then go back to our consult groups. To other people that we know are working with teams and say, ‘You know what, I’ve been doing this. This is whaťs been happening. Iťs been helpful for my clients. I am getting compensated for it.’”).

## Study 2

### Methods

#### Participants.

Adolescents who were identified as needing treatment for depression by a behavioral health provider who participated in Study 1 were recruited to participate in the study, along with their parents. Inclusion criteria were as follows: (a) adolescent age 12–17, (b) adolescent diagnosed with a depressive disorder (Major Depressive Disorder, Persistent Depressive Disorder, Other Specified Depressive Disorder) by their behavioral health provider, (c) at least one parent/caregiver willing to participate in the study, and (d) parent and adolescent English-speaking/reading/writing ability at a level to provide informed consent/assent and complete questionnaires. Adolescents were excluded if they reported active suicidal ideation with a plan and/or intent and/or the provider assessed the adolescent to be in need of a higher level of care than outpatient care. These adolescents were excluded because the treatment options recommended by the CDSS-YD are outpatient-level treatments.

Six adolescents (mean age = 14.00, SD = 1.41) and their parents/caregivers participated in the study. Adolescent sex assigned as birth was as follows: three female, two male, and one prefer not to answer. One adolescent identified their gender as female, one identified as male, two identified as transgender male, one identified as non-binary, and one identified as gender fluid. All adolescents were white and were not Hispanic or Latino. All parent participants were biological mothers. Mean household income was $82,500 (SD = $52,360.92).

Seven of the eight behavioral health providers who participated in Study 1 volunteered to participate in Study 2. The eighth provider did not participate because she was no longer seeing adolescent-aged patients.

#### Study Procedures.

Following collection of informed consent and assent, adolescents and parents were provided with the web link to complete the CDSS-YD self- and parent-report measures. At their next scheduled appointment with the behavioral health provider, the provider administered the CDSS-YD provider measures. The provider then reviewed the CDSS-YD treatment recommendation page with the family as part of their treatment planning discussion. Following completion of the clinic visit, the provider completed a survey about the process and outcome of using the CDSS-YD. The parent and adolescent also completed a survey about their attitudes regarding the use of the CDSS-YD.

#### CDSS-YD Provider Training.

Behavioral health providers were trained by a clinical psychologist to utilize the CDSS-YD. Training, which included watching a 20-minute video and attending a 1-hour live training conducted via a web-based video platform, included background on the development of the CDSS-YD, training in the administration of the provider-administered CDSS-YD measures, and training in the use of the CDSS-YD to inform treatment planning discussions with families. Providers were also given sample scripts to introduce and discuss the CDSS-YD recommended treatment, answer common questions, and engage in shared-decision making to formulate a treatment plan.

#### Measures.

##### CDSS-YD Outcome.

Following the clinical encounter with the family, providers documented whether they recommended the CDSS-YD-recommended treatment and whether parents and adolescents indicated they would like to initiate the CDSS-YD-recommended treatment. If the provider and/or family indicated they would not initiate the recommended treatment, providers documented the reasons why the provider, adolescent, and/or parent did not want to initiate the CDSS-YD-recommended treatment, the alternative treatment selected, and reasons for selecting that treatment.

##### CDSS-YD Attitudes.

Parents and adolescents rated the extent to which they liked the CDSS-YD, found it easy to understand, helpful, and relevant to their lives using a 5-point Likert scale (1 = completely disagree, 3 = neither disagree nor agree, 5 = completely agree). Parents, adolescents, and providers were also asked open-ended questions regarding what they liked about the CDSS-YD, what they did not like, and what would make the CDSS-YD better.

### Results

#### CDSS-YD Treatment Selection Outcome.

The treatment selection outcomes following use of the CDSS-YD are listed in Table 2. One family out of six did not chose to proceed with a treatment that was recommended by the CDSS-YD. In this case, the CDSS-YD recommended medication only and the provider and family preferred and chose combination treatment because the adolescent wanted a therapist to talk to and the provider and parent felt the adolescent could use as much help as possible and would benefit from receiving both therapy and medication.

#### CDSS-YD Attitudes.

Parents’ and adolescents’ mean ratings on the CDSS-YD Attitudes measure are listed in Table 3. On average, they reported that they liked the CDSS-YD, found it easy to understand, and reported that it had information that was helpful and relevant to their lives.

Parents reported liking that the CDSS-YD was concise and easy to understand and they liked that it helped direct them towards a treatment that was likely to be helpful for their child. Adolescent reported that they liked that it helped direct them towards a treatment that would be best for them personally. Providers reported liking that the treatment recommendation page was detailed, it helped provide some structure to the treatment planning process, and made it easy for them to explain the treatment recommendations to the family. A concern raised by both parents and providers was that it sometimes took a few sessions to complete all the CDSS-YD components and treatment planning discussion, which delayed the process of coming to a decision. Parents requested a paper copy of the CDSS-YD treatment recommendation page. Adolescents did not report any concerns about the CDSS-YD.

## Discussion

The current study collected mixed-method feedback from adolescents, parents, and behavioral health providers on a computationally-based CDSS that guides personalized treatment planning for youth with depression. Overall, all stakeholder groups liked the CDSS-YD. They found it easy to understand and useful for making treatment decisions. This was true for providers and families who viewed the CDSS-YD during a focus group, as well as for those who used it during a clinical encounter. They perceived the CDSS-YD to provide clarity and direction for engaging in treatment planning, which can otherwise often feel like an ambiguous or “trial and error” process. Providers reported liking that the CDSS-YD helped provide some structure to the treatment planning process and made it easy for them to explain the treatment recommendations to the family. Parents and providers particularly liked that the CDSS-YD was developed from research and that the treatment recommendation was based on objective data, as opposed to an opinion, which could be perceived as biased. Parents also reported that providers’ use of the CDSS-YD would increase their perception of the providers’ expertise because it would indicate they were knowledgeable about the most recent science. Of note, some of the negative beliefs and attitudes towards the CDSSs that were identified in other studies were not identified regarding the CDSS-YD, including the belief that the use of CDSS would reduce providers’ professional autonomy or interfere with the provider-patient therapeutic relationship. In fact, all stakeholder groups viewed the use of the CDSS-YD as a way of strengthening the therapeutic relationship.

While parents and adolescents found the CDSS-YD to be beneficial for treatment planning, they also clearly viewed it as a tool that needed to be used with a provider. They viewed the provider as central to understanding and trusting the CDSS-YD and to coming to a treatment decision. Both parents and youth noted the need for providers to answer questions, give additional information, and provide clarification. They also reported that they would be more likely to trust the CDSS-YD recommendation if their provider also agreed with it. This was particularly the case for youth, who actually expressed having more trust in their providers than in a new mathematical algorithm. They viewed their providers as more trustworthy because of their perceived extensive training and experience. Using the CDSS-YD with a provider is also important given parents’ and adolescents’ desire to understand how their questionnaire responses led to their particular treatment recommendation. The idea that the recommendation came from a statistical equation that included all of the questionnaire scores was more difficult for them to understand and required additional explanation. The critical role of the provider in using the CDSS-YD speaks to the need for effective training so that providers are well-equipped to use, explain, and answer questions about the CDSS-YD.

A theme around adolescents’ desire for privacy also emerged. Some adolescents expressed discomfort with their parents seeing their questionnaire results because of concern about how their parents might react or the potential for it to cause their parents to make unwanted treatment decisions for them. These adolescents expressed a preference for parents and adolescents to review the CDSS-YD results with the provider separately so the adolescents could process the information and form their own thoughts with their provider on their own and not experience the anticipated discomfort of observing the conversation between the provider and their parents. This identifies the importance of training providers to attend to and balance adolescents’ growing desire and need for privacy and autonomy in making treatment decisions with parents ultimately having final decision on their child’s medical care.

Providers who were trained to implement the CDSS-YD were able to use it successfully with a relatively brief training. All providers and families who used the CDSS-YD during a clinical encounter reported using the CDSS-YD information to inform their treatment decision. In several cases, the CDSS-YD suggested that CBT, medication, and combination treatment were likely to be equally effective, which provided the opportunity for family preference. In one case, the family did not move forward with the CDSS-YD recommended treatment – the CDSS-YD recommended medication only and the family and provider felt the adolescent would also benefit from the addition of CBT. The providers were trained to view and present the CDSS-YD as a tool that provides some information for the provider and family to consider and to inform a shared decision-making process with families, but not a treatment mandate. The fact that one family and provider did deviate from the CDSS-YD recommendation suggests that they did feel empowered to use the CDSS-YD flexibly.

All families ultimately expressed intention to initiate combination treatment for their adolescent. It is not known whether families did move forward with initiating that treatment. Given families’ historically low rates of mental health treatment initiation (19), and families’ view that the CDSS-YD provided clarity and personalization to a treatment planning process that can often feel like a trial-and-error approach, future research might examine the impact of CDSS-YD use on treatment initiation and ongoing engagement. In this way, a computationally-based CDSS for youth depression may the potential to have a positive impact on treatment outcomes via two pathways: via a more effective personalized treatment recommendation and via a positive impact on families’ initiation and engagement in services.

The results of this study have significant implications for the development of larger-scale implementation efforts for the CDSS-YD specifically and potentially other CDSSs more broadly. Overall, the attitudinal barriers reported in other studies of CDSSs were not reported for the CDSS-YD. Parents, adolescents, and providers all had positive attitudes towards the CDSS-YD. Instead, the primary identified barrier to implementation was a system-level barrier. Clinic productivity metrics were perceived to have a significant negative impact on having the time and energy to integrate the CDSS-YD into practice, and it was perceived to negatively impact providers, patients, and parents alike. Providers reported that while, in theory, they would be eager to learn to use new tools that could help their patients, the time and mental labor needed to learn something new could feel prohibitive on top of providers’ already demanding productivity expectations and experience of burnout. Similarly, stakeholders perceived there to be insufficient time in the clinical workflows for families to complete of the questionnaires that are essential for CDSS-YD use.

The providers noted that the provision of protected time for training, practice, and use; and integration of the CDSS-YD into the medical record would help reduce burden and facilitate implementation. Protected time was also viewed as a facilitator to families’ successful completion of the questionnaires needed for CDSS-YD use. Additional time was recommended not only for families to complete the questionnaires, but for providers to be able to explain to families why careful and complete responses to the questionnaires was needed for the CDSS-YD to work. Families reported that they are frequently asked to complete questionnaires for medical appointments which do not appear to be connected to the care they receive, leading to survey fatigue and not viewing the questionnaires to be relevant to their care. They expressed the importance of clearly explaining prior to the appointment the significance of the questionnaires for informing the development of their plan of care.

While providers did not report attitudinal barriers to CDSS-YD use, they did report that positive messaging about the CDSS-YD could be helpful. They reported that providers might feel more motivated to engage in learning and use of the CDSS-YD if they heard positive feedback from colleagues and families. They suggested starting first with providers who volunteer and having those providers report their experiences back to the others.

In sum, the results of this study support the feasibility and acceptability of a machine-learning based CDSS for youth depression. It has the potential to be a widely accepted and useful tool for personalized treatment planning. Successful implementation will require addressing the system-level barrier of having sufficient time and energy to integrate the CDSS-YD into practice.

## Figures and Tables

**Table 1.** Key Themes Regarding Provider, Parent, and Adolescent Attitudes Toward the CDSS-YD

Themes	Subthemes	Exemplar Quotations
Providers, parents, and adolescents viewed the CDSS-YD as helpful for treatment.	Providers, parents, and adolescents viewed the CDSS-YD as an informative and useful tool for personalized treatment planning.	“I think iťd be helpful because sometimes even the parents or the team donť necessarily know what might be helpful in their situation. So this would kind of be a nice way of, ‘Well, leťs do this assessment tool we have and see what, based YD on your answers, where the benefit might be.’” -Provider “I guess for me just thinking if I had this, then perhaps we would have saved my daughter six months’ worth of therapy that was not going to be helpful. ” - Parent “I think iťs helpful. You know, you might be in a situation where you’re not real big on medicine and you might think that all clinicians that you see are going to be recommending medication. So if you take this survey, and based on your answers the survey is pointing them in this direction, iťs kind of like an affirmation. It would kind of be like getting more of just one single person’s opinion as to what the treatment plan should be” - Parent “I think it’s nice just because it has an answer right there. Like you took this poll, and it’s not like you’re dissecting data, it’s just it gives you an answer of what a possible solution is that would be very helpful based on data.” - Adolescent
	Providers, parents, and adolescents believed that use of the CDSS-YD could foster a stronger therapeutic relationship	“And I was just going to say, I think data always helps kind of give families trust in you. Being you’re not just throwing this recommendation out without having good knowledge behind it. And I think just having this. This is kind of what those showed us and there’s research to kind of help prove behind that it is effective. I think that can always help in relationships.” - Provider “I think anytime you’re working with teens, asking for their input is always a good building block. Because often, I think they feel that they donť really have a say in a lot of things, and iťs not their choice. So I think that parťs really nice.” - Provider “It opens up like the conversation… the provider, the adolescent, and the parent.” - Parent “I think it would help because it just sort of like throws everything out in the open. Like it just is like ‘Yeah, you do have a problem with this and this and this.’ It’s not just like you kind of avoiding answering questions if you answer truthfully on the survey. I think it would help, especially if you are talking about it separately from your parents, being able to discuss the results or treatment options. I just think it would help.” -Adolescent
Providers, parents, and adolescents differed in their views of the trustworthiness of the CDSS-YD.	Providers and parents viewed the CDSS-YD as trustworthly because it is research-based and the treatment recommendation is data-driven.	“The fact that it was based on a study for me helped increase it.” -Provider “I think data always helps give families trust in you - being you’re not just throwing this recommendation out without having good knowledge behind it.” -Provider “I’m a data person so if you give me data and studies and say this came from studies of a lot of people and this is how this tool came about, I’m more apt to agree to a treatment recommendation than to say well we’ll try therapy first.” - Parent
	Use of the CDSS-YD increased parents’ trust in the provider and their perception of provider expertise.	“I think it would affirm that providers know that these particular sets of answers lead to this conclusion instead of just tossing out ‘Oh, leťs try this or whatever.’ So in that respect, it would make me feel more comfortable that they really know that they’re talking about.” - Parent
	Youth viewed the CDSS-YD treatment recommendation as less trustworthy than a provider recommendation.	“Personally probably [I’d trust] a doctor just because there’s always room for human error but doctors have been doing it for a long time and they’ve had training and everything. I mean, personally I think I would trust a doctor’s opinion more.” - Adolescent “Doctors have been doing this for decades and decades. And this algorithm has been at it for I don’t know how many years but I can’t imagine it being as long.” - Adolescent
Parents and adolescents saw their providers as having a critical role in the use the CDSS-YD.	The provider’s opinion of the CDSS-YD treatment recommendation was a facilitator of families’ trust.	“How much the therapist that you’re going over the treatment with. I mean, if they’re putting trust in it, then iťs more likely that you’re going to be trusting the recommendation.” - Parent “I think if a doctor was like yeah I agree with this recommendation based on your personal history and how you answered the questions I think that would make it better.” - Adolescent
	It is important for the CDSS-YD to be used in the context of a discussion with the provider.	“The doctor is there and can break it down more too.” - Parent “And having someone there to say hey, what does this mean? What does this mean for us? Or what’s common, right? So I think having that interaction with a provider, a professional what have you I think is, for me what would make it better which is already happening.” - Parent “The section provides a lot of information but it has you deal with it on your own. Unless you’re a super good medical professional that knows exactly what you’re doing, it’s going to be hard to make a judgement off of that.” - Adolescent
Providers, parents, and adolescents expressed a desire to understand how the questionnaire responses informed the CDSS-YD’s treatment recommendation.		“I think it might be helpful to maybe even have a brief description of why those ones are recommended.” - Provider “And for me this is not really clear…the reason why you’re recommending your treatment” - Parent “Even though it tells me the statistics on me, it doesn’t tell me how they came to the conclusion.” - Adolescent
Adolescents expressed discomfort with sharing their questionnaire results with the parents, and they expressed a desire for privacy when reviewing the CDSS-YD results with the provider.		“And I think that my parents specifically would look at it and then decide something for themselves and be like ‘Oh, you have inattention; you should have a tutor.’ It isn’t necessarily what I would need.” - Teen “It’s just kind of uncomfortable for me, especially I don’t really like telling them how I’m feeling. I know it’s necessary, but just, like, being there for that is kind of just weird to me.” - Adolescent “I think personally I would just rather have to look at that separately so I wouldn’t have to deal with that information, but also have to deal with my parents’ reactions to it, I guess. I feel like that would be a lot harder to do.” - Adolescent

**Table 2.** CDSS-YD Treatment Selection Outcomes

Participant	CDSS-YD Recommended Treatment	Provider Recommended Treatment	Parent Preferred Treatment	Adolescent Preferred Treatment	Treatment Chosen by Family
1	COMB	COMB	COMB	COMB	COMB
2	CBT, MED, or COMB	COMB	COMB	COMB	COMB
3	MED	COMB	COMB	COMB	COMB
4	CBT, MED, or COMB	COMB	COMB	MED	COMB
5	CBT, MED, or COMB	COMB	COMB	COMB	COMB
6	CBT, MED, or COMB	COMB	COMB	COMB	COMB

Note. CBT = Cognitive Behavioral Therapy, COMB = Combination Treatment, MED = Medication

**Table 3.** Parent and Adolescent Attitudes Regarding the CDSS-YD

	Parent M (SD)	Adolescent M (SD)
I like the treatment planning tool.	4.00 (.00)	3.80 (.84)
The treatment planning tool was easy to understand.	4.20 (.45)	4.20 (.84)
The treatment planning tool had information that was helpful to me.	3.60 (.89)	3.60 (.89)
The treatment planning tool had information that was relevant to my (my teen’s) life.	4.00 (.70)	3.40 (.89)

## Abbreviations

CBT: Cognitive Behavioral Therapy
CDSS: Clinical Decision Support System
CDSS-YD: Clinical Decision Support System for Youth Depression
COMB: Combination Treatment
MED: Medication
TADS: Treatment of Adolescents with Depression Study
